# Commentary: Rational Adaptation in Lexical Prediction: The Influence of Prediction Strength

**DOI:** 10.3389/fpsyg.2021.735849

**Published:** 2021-08-24

**Authors:** Mante S. Nieuwland

**Affiliations:** ^1^Neurobiology of Language Department, Max Planck Institute for Psycholinguistics, Nijmegen, Netherlands; ^2^Donders Institute for Cognition, Brain and Behaviour, Nijmegen, Netherlands

**Keywords:** expectation adaptation, rational adaptation, Bayesian adaptation, probabilistic prediction, predictive cue validity, prediction error

People sometimes predict specific words during language comprehension. It is thought that while correct predictions benefit word recognition and incremental comprehension, incorrect predictions can incur a cognitive processing cost, possibly because people have to inhibit the word representations they had activated ahead of time (e.g., Ness and Meltzer-Asscher, 2018). According to the “rational adaptation hypothesis” (e.g., Kuperberg and Jaeger, 2016), people balance these costs and benefits by rationally adapting lexical predictions to the estimated probability of prediction disconfirmation (error). Increased probability of disconfirmation leads to weaker predictions, which, in turn, may reduce the processing costs incurred by prediction failure. In support of this hypothesis, Ness and Meltzer-Asscher (2021, from hereon NMA2021) report a clever and impressive study with two large-scale behavioral experiments and largely pre-registered analyses. The current commentary critically reviews these analyses and performs re-analyses which show that their data, in fact, do *not* support the rational adaptation hypothesis.

## Summary of NMA2021

Participants gave speeded congruency judgments to prime-target word-pairs. Word-pairs were of three types^1^ based on whether the prime strongly suggested a likely target and whether the target was predictable given the prime, corresponding to the pre-rated “constraint” and “cloze” value^2^, respectively. Assuming that constraining primes caused participants to predict the most likely target, trials could involve disconfirmed prediction (High Constraint prime, Low Cloze target: High-Low trials), confirmed prediction (High-High) or no prediction (Low-Low). To investigate adaptation, NMA2021 manipulated the proportion of High-Low/Low-Low filler trials between participants in three lists (High-Low list: 60/0, Low-Low list: 0/60, mixed list: 30/30).

NMA2021 pre-registered a statistical Base model (1) of log-transformed reaction time as a function of trial type, trial number, list and all interactions. They reported that the difference between High-Low and Low-Low trials (taken as indexing the relative costs of disconfirmed prediction) was smaller in the High-Low list than in the Low-Low list. However, this result in itself does not conclusively support rational adaptation, and evidence for the crucial three-way interaction with trial number was insufficient.

1) Base model: log RT ~ Trial type ^*^ Trial number ^*^ List

**Figure 1:**
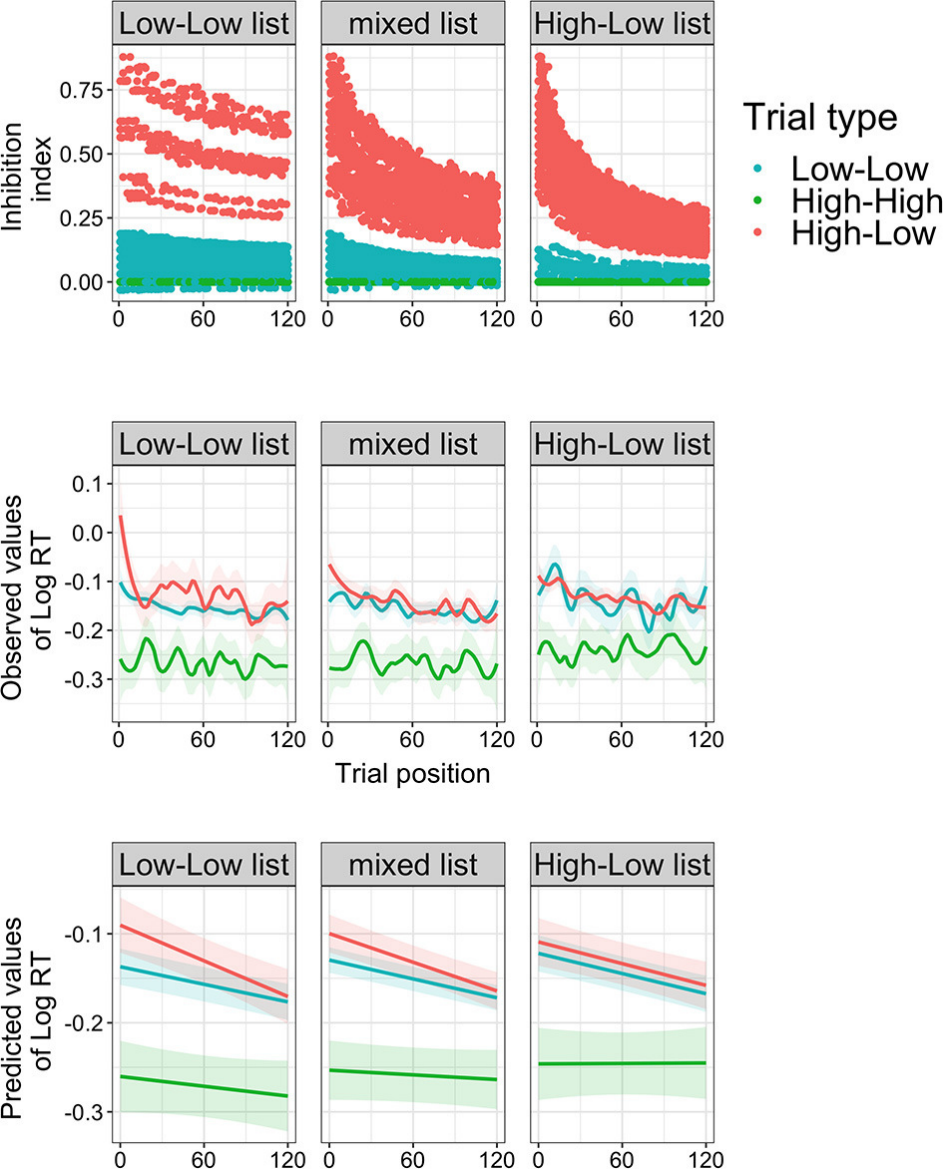
Model output and reaction time (log RT) results from NMA2021's Experiment 2. Top row: output of the rational adaptor model (inhibition index) for each trial type and trial list at each trial position in the experiment (adapted from NMA2021). Middle row: observed reaction times (regression fitted with ‘Local Polynomial Regression Fitting’ and *t*-value based confidence bounds). Bottom row: predicted reaction times from the linear regression Base model with which NMA2021 tested for a three-way interaction between trial position, trial list, and trial type. N.B. Like NMA2021's graphs, these graphs use both critical and filler trials, but results and graphs for critical trials only are available on OSF.

NMA2021 further pre-registered a Bayesian adaptation model that weighted prediction error (PE: prime constraint minus target cloze) with the estimated probability of prediction confirmation given the history of confirmation at each given trial (μ), with the formula: PE^*^μ. The model output was dubbed “inhibition index,” the assumed processing costs from inhibiting disconfirmed predictions. The inhibition index is higher overall for High-Low trials than for Low-Low trials and High-High trials (Figure 1, top row). Displaying rational adaptation, the inhibition index of High-Low trials rapidly decreases with trial position in the High-Low list, slower in the mixed list and slowest in the Low-Low list.

Crucially, the inhibition index was a statistically significant predictor when added to the Base model (2), and in an Adaptation model (3) along with cloze. Moreover, the Adaptation model yielded a better fit than models that weighted PE by either trial number/position (4) or by HL/LL trial counts (5). NMA2021 therefore concludes that “the assumptions of the Bayesian adaptation model indeed increase its explanatory power, relative to other models including the basic information entered into its calculations, but without its additional assumptions.”

2) Base model with inhibition: log RT ~ Trial Type ^*^ Trial Number ^*^ List + Inhibition

3) Adaptation model: log RT ~ Inhibition Index + Cloze

4) Position model: log RT ~ PE ^*^ Trial Position

5) Count model: log RT ~ PE ^*^ HL trial count ^*^ LL trial count.

## Critique

NMA2021's claims regarding rational adaptation rest on two findings. First, the Base model showed that prediction disconfirmation costs (High-Low minus Low-Low) were smaller in the High-Low list compared to the Low-Low list. However, their analysis erroneously included filler trials, which may have generated spurious results. Moreover, the Base model suffered from massive multicollinearity (e.g., Alin, 2010). After z-transforming List and Trial Position, analysis of critical trials alone did not support NMA2021's claims.

Second, NMA2021's conclusions rest on the behavior and statistical significance of the inhibition index. However, the observed responses and predicted values from the Base model (Figure 1, middle and bottom row, respectively) were not consistent with the inhibition index. Differences between High-Low and Low-Low trials occur in the initial trials of the Low-Low list, but not in the High-Low list. So, why did the inhibition index yield an effect at all? This is because the inhibition index is linearly related to other predictors and competes for explained variance, which is why multicollinearity remained problematic for the Base model with inhibition even after z-transforming List and Trial Position. As visible from NMA2021's, adding the inhibition index weakens previously strong effects of trial type and trial position. Importantly, the inhibition index enjoys an unfair advantage, not because of μ, but because it relies on PE, a continuous measure that is closer to the actual manipulation than the categorical predictor trial type.

Comparisons between the Adaptation model and the Position and Count models are also problematic. Only the Adaptation model includes Cloze, even though the argument for including Cloze applies to all models, namely to account for facilitatory effects of correct predictions which are not captured by PE. Because Cloze is a strong predictor of reaction time (Figure 1; see also Smith and Levy, 2013), this model comparison is “unfair” because a model with Cloze will outperform a model without. This is easily addressed: a Position model with Cloze (6) outperforms the Adaptation model [$\chi_{\left(2\right)}^{2}$ = 53.6, *p* < 0.001], and the original Position model outperforms the Adaptation model without Cloze (7), [$\chi_{\left(2\right)}^{2}$ = 37.96, *p* < 0.001]. The same goes for a Count model^3^. Of note, the Adaptation model is possibly suboptimal because it does not capture separate variance of PE and μ. But even compared to an alternative Adaptation model that does (8), the simpler Position model with Cloze still fits the data as well, and even better when random slopes are retained.

(6) Position model with Cloze: log RT ~ PE ^*^ Trial Position + Cloze

(7) Adaptation model without Cloze: log RT ~ Inhibition Index

(8) Alternative adaptation model: log RT ~ PE ^*^ μ + Cloze

Finally, NMA2021's conclusions are refuted not just by these exploratory analyses, but also by their own pre-registered analyses. NMA2021 erroneously compared the Position and Count models with the Base model with inhibition, not with the Adaptation model as per their pre-registration. In the pre-registered comparison, the Adaptation model was outperformed by the Count model (Experiment 2) and by the Position model (Experiment 1).

## Conclusion

The current reanalyses show that NMA2021's conclusions do not uphold. Responses changed during the experiment but not in a way that supported rational adaptation to prediction disconfirmation. Moreover, the key assumption of their Bayesian adaptation model, weighted prediction error, does not *increase* and may even *decrease* its explanatory power compared to other relevant models. Therefore, prediction disconfirmation was not a crucial trigger for adaptation. With related conclusions (Delaney-Busch et al., 2019) similarly debunked by Nieuwland (2021), the rational adaptation hypothesis of prediction is now left on shaky ground. Linguistic prediction may be more robust to changes in statistical regularities in the local environment than is sometimes thought.

One key question remains: why was the response time difference between High-Low and Low-Low trials large at the beginning of the Low-Low list, but not the High-Low list? This pattern is possibly a spurious result from having different subjects per list or low trial numbers, or could be caused by another variable that was not included in the statistical models. Regardless, responses to low cloze targets sped up in all lists, which may have impacted slowest responses most thereby yielding an interaction pattern (see also Prasad and Linzen, 2019). While such changes are not “rational” within the rational adaptation framework, they can be viewed as rational in a colloquial sense because they nevertheless reflect adaptation to the task environment (see also Nieuwland, 2021).

Without a doubt, NMA2021 deserves credit for pre-registering analyses and making data and scripts publicly available. But NMA2021 also demonstrates that pre-registration and data availability during peer review are no panacea (see also Szollosi et al., 2020). Pre-registered analyses can be misconceived, and such problems and analysis errors can be overlooked by researchers, peer reviewers and journal editors alike. This merely strengthens the case for scientific transparency and open data, to allow for post-publication re-analyses such as reported here and in Nieuwland (2021).

## Author Contributions

The author confirms being the sole contributor of this work and has approved it for publication.

## Conflict of Interest

The author declares that the research was conducted in the absence of any commercial or financial relationships that could be construed as a potential conflict of interest.

## Publisher's Note

All claims expressed in this article are solely those of the authors and do not necessarily represent those of their affiliated organizations, or those of the publisher, the editors and the reviewers. Any product that may be evaluated in this article, or claim that may be made by its manufacturer, is not guaranteed or endorsed by the publisher.

## Footnotes

^1^In Experiment 2 (English), the focus of this commentary. Similar arguments apply to Experiment 1 (Hebrew, 2 word-pair types), detailed results are available on OSF.

^2^In a Cloze completion test, an independent sample of participants completed prime words with a subsequent word. Target cloze was the percentage of participants using the target, prime constraint was the highest cloze value obtained for that prime.

^3^NMA2021's Count model suffers from multicollinearity because the HL and LL trial counts are correlated. But a model that avoids multicollinearity by counting HL and LL trials together also outperforms the Adaptation model.
